# (*E*)-4-(2-Chloro­benzyl­ideneamino)-3-(2-chloro­phen­yl)-1*H*-1,2,4-triazole-5(4*H*)-thione–(*E*)-1,5-bis­(2-chloro­benzyl­idene)thio­carbonohydrazide–methanol (1/1/1)

**DOI:** 10.1107/S1600536809053203

**Published:** 2009-12-16

**Authors:** Qingliang Guo

**Affiliations:** a Department of Chemistry and Environmental Science, Taishan University, 271021 Taian, Shandong, People’s Republic of China

## Abstract

In the title compound, C_15_H_12_Cl_2_N_4_S·C_15_H_10_Cl_2_N_4_S·C_2_H_6_O, the two chloro­phenyl rings of the triazole derivative form dihedral angles of 65.7 (2) and 44.2 (2)° with the triazole ring. In the thio­carbonohydrazide derivative, the dihedral angle between the two chloro­phenyl rings is 5.4 (2)°.  In the crystal, the triazole, thio­carbonohydrazide and methanol mol­ecules are linked by N—H⋯O, N—H⋯S and O—H⋯S hydrogen bonds, forming a hexa­meric unit.

## Related literature

For general background to Schiff bases, see: Ren *et al.* (1999 ▶); Yang *et al.* (2005 ▶); Sen *et al.* (1998 ▶); Xia *et al.* (2007 ▶). For the biological activity of Schiff bases, see: Liang (2003 ▶); Bacci *et al.* (2005 ▶).
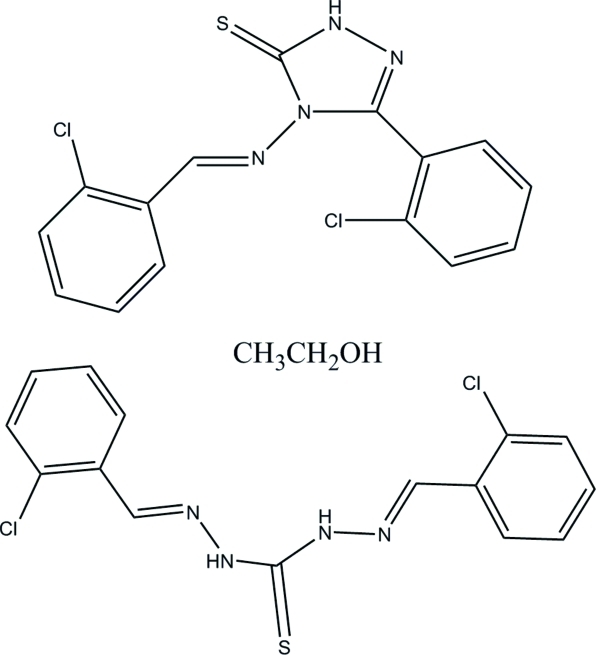


## Experimental

### 

#### Crystal data

C_15_H_10_Cl_2_N_4_S·C_15_H_12_Cl_2_N_4_S·C_2_H_6_O
*M* *_r_* = 746.54
Triclinic, 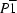

*a* = 7.7086 (6) Å
*b* = 10.9999 (9) Å
*c* = 20.9827 (16) Å
α = 94.678 (1)°
β = 92.083 (1)°
γ = 99.851 (1)°
*V* = 1744.8 (2) Å^3^
*Z* = 2
Mo *K*α radiation
μ = 0.50 mm^−1^
*T* = 293 K
0.35 × 0.26 × 0.23 mm

#### Data collection

Bruker SMART APEX diffractometer
Absorption correction: multi-scan (*SADABS*; Sheldrick, 1996 ▶) *T*_min_ = 0.845, *T*_max_ = 0.894
9324 measured reflections
6154 independent reflections
3724 reflections with *I* > 2σ(*I*)
*R*_int_ = 0.026

#### Refinement

*R*[*F* ^2^ > 2σ(*F* ^2^)] = 0.046
*wR*(*F* ^2^) = 0.111
*S* = 1.01
6154 reflections
425 parameters
H-atom parameters constrained
Δρ_max_ = 0.28 e Å^−3^
Δρ_min_ = −0.25 e Å^−3^

### 

Data collection: *SMART* (Bruker, 2005 ▶); cell refinement: *SAINT* (Bruker, 2005 ▶); data reduction: *SAINT*; program(s) used to solve structure: *SHELXS97* (Sheldrick, 2008 ▶); program(s) used to refine structure: *SHELXL97* (Sheldrick, 2008 ▶); molecular graphics: *SHELXTL* (Sheldrick, 2008 ▶); software used to prepare material for publication: *SHELXL97*.

## Supplementary Material

Crystal structure: contains datablocks I, global. DOI: 10.1107/S1600536809053203/ci2984sup1.cif

Structure factors: contains datablocks I. DOI: 10.1107/S1600536809053203/ci2984Isup2.hkl

Additional supplementary materials:  crystallographic information; 3D view; checkCIF report

## Figures and Tables

**Table 1:** Hydrogen-bond geometry (Å, °)

*D*—H⋯*A*	*D*—H	H⋯*A*	*D*⋯*A*	*D*—H⋯*A*
N2—H2⋯O1	0.86	2.20	2.978 (4)	150
N6—H6⋯S1^i^	0.86	2.41	3.261 (3)	170
N3—H3⋯S2^ii^	0.86	2.56	3.410 (2)	169
O1—H1⋯S1^iii^	0.82	2.65	3.413 (3)	156

Symmetry codes: (i) 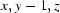; (ii) 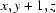; (iii) 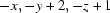.

**Fig. 1.**
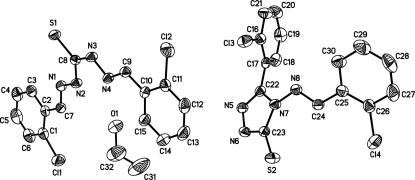
The asymmetric unit of the title compound, with atom labels and 30% probability displacement ellipsoids for non-H atoms. H atoms have been omitted for clarity.

## supplementary crystallographic information

### Comment

The synthesis and structural investigation of Schiff base compounds have attracted much attention due to their interesting structures and potential applications. Some of them have biological activities (Liang, 2003; Bacci *et al.*, 2005). They also play an important role in the development of coordination chemistry as well as inorganic biochemistry, catalysis and optical materials (Ren *et al.*, 1999; Yang *et al.* 2005; Sen *et al.* 1998).

The triazole ring forms dihedral angles of 65.7 (2) and 44.2 (2)°, respectively, with the C16-C21 and C25-C30 rings. The dihedral angle between the C1-C6 and C10-C15 rings is 5.4 (2)°. Two triazole, two thiocarbonohydrazide and two methanol molecules are linked by N—H···O, N—H···S and O—H···S hydrogen bonds to form a hexamer.

### Experimental

The Schiff base compound was synthesized according to the modified method of Xia *et al.* (2007). A mixture of (2-chlorophenyl)methanamine and thiourea in methanol (30 ml) was refluxed for 3 h and filtered. The filtrate was placed for sevaral days yielding colourless block-shaped crystals. (yield 79%). Elemental analysis: Calculated for C_32_H_28_Cl_4_N_8_OS_2_: C 51.48, H 3.78, N 15.01; found: C 51.51, H 3.49, N 15.13.

### Refinement

The H atoms were found in a difference map, then placed in idealized positions (C-H = 0.93–0.97 Å, N-H = 0.86 Å and O-H = 0.82 Å), and refined using a riding model, with U_iso_(H) = 1.2*U*_eq_(C,N) and 1.5*U*_eq_(O,C_methyl_).

### Crystal data

**Table:**

C_15_H_10_Cl_2_N_4_S·C_15_H_12_Cl_2_N_4_S·C_2_H_6_O	*Z* = 2
*M**_r_* = 746.54	*F*(000) = 768
Triclinic, *P*1	*D*_x_ = 1.421 Mg m^−^^3^
Hall symbol: -P 1	Mo *K*α radiation, λ = 0.71073 Å
*a* = 7.7086 (6) Å	Cell parameters from 1667 reflections
*b* = 10.9999 (9) Å	θ = 2.2–20.5°
*c* = 20.9827 (16) Å	µ = 0.50 mm^−^^1^
α = 94.678 (1)°	*T* = 293 K
β = 92.083 (1)°	Block, colourless
γ = 99.851 (1)°	0.35 × 0.26 × 0.23 mm
*V* = 1744.8 (2) Å^3^	

### Data collection

**Table:**

Bruker SMART APEX diffractometer	6154 independent reflections
Radiation source: fine-focus sealed tube	3724 reflections with *I* > 2σ(*I*)
graphite	*R*_int_ = 0.026
φ and ω scans	θ_max_ = 25.1°, θ_min_ = 1.0°
Absorption correction: multi-scan (*SADABS*; Sheldrick, 1996)	*h* = −7→9
*T*_min_ = 0.845, *T*_max_ = 0.894	*k* = −12→13
9324 measured reflections	*l* = −24→24

### Refinement

**Table:**

Refinement on *F*^2^	Primary atom site location: structure-invariant direct methods
Least-squares matrix: full	Secondary atom site location: difference Fourier map
*R*[*F*^2^ > 2σ(*F*^2^)] = 0.046	Hydrogen site location: inferred from neighbouring sites
*wR*(*F*^2^) = 0.111	H-atom parameters constrained
*S* = 1.01	*w* = 1/[σ^2^(*F*_o_^2^) + (0.0365*P*)^2^ + 0.4564*P*] where *P* = (*F*_o_^2^ + 2*F*_c_^2^)/3
6154 reflections	(Δ/σ)_max_ = 0.001
425 parameters	Δρ_max_ = 0.28 e Å^−^^3^
0 restraints	Δρ_min_ = −0.25 e Å^−^^3^

### Special details

**Table:**

Geometry. All e.s.d.'s (except the e.s.d. in the dihedral angle between two l.s. planes) are estimated using the full covariance matrix. The cell e.s.d.'s are taken into account individually in the estimation of e.s.d.'s in distances, angles and torsion angles; correlations between e.s.d.'s in cell parameters are only used when they are defined by crystal symmetry. An approximate (isotropic) treatment of cell e.s.d.'s is used for estimating e.s.d.'s involving l.s. planes.
Refinement. Refinement of *F*^2^ against ALL reflections. The weighted *R*-factor *wR* and goodness of fit *S* are based on *F*^2^, conventional *R*-factors *R* are based on *F*, with *F* set to zero for negative *F*^2^. The threshold expression of *F*^2^ > σ(*F*^2^) is used only for calculating *R*-factors(gt) *etc*. and is not relevant to the choice of reflections for refinement. *R*-factors based on *F*^2^ are statistically about twice as large as those based on *F*, and *R*- factors based on ALL data will be even larger.

### Fractional atomic coordinates and isotropic or equivalent isotropic displacement parameters (Å^2^)

**Table:**

	*x*	*y*	*z*	*U*_iso_*/*U*_eq_	
S1	0.25610 (13)	1.26698 (8)	0.43362 (4)	0.0574 (3)	
S2	0.40630 (12)	0.14705 (9)	0.24096 (4)	0.0614 (3)	
Cl1	0.15231 (15)	0.94451 (10)	0.71997 (4)	0.0836 (3)	
Cl2	0.48468 (13)	0.77336 (9)	0.21104 (4)	0.0738 (3)	
Cl3	0.21316 (12)	0.52305 (8)	0.10925 (5)	0.0689 (3)	
Cl4	0.22413 (15)	−0.15631 (9)	0.07317 (5)	0.0861 (3)	
O1	0.1682 (4)	0.7954 (3)	0.52080 (14)	0.0837 (8)	
H1	0.0608	0.7901	0.5211	0.126*	
N1	0.1820 (3)	1.1229 (2)	0.54344 (12)	0.0487 (7)	
N2	0.2441 (3)	1.0597 (2)	0.49247 (11)	0.0472 (7)	
H2	0.2608	0.9847	0.4944	0.057*	
N3	0.3341 (3)	1.0539 (2)	0.39024 (11)	0.0506 (7)	
H3	0.3534	1.0877	0.3550	0.061*	
N4	0.3618 (3)	0.9349 (2)	0.39441 (11)	0.0468 (6)	
N5	0.0553 (4)	0.3611 (2)	0.26242 (12)	0.0529 (7)	
N6	0.1796 (3)	0.2949 (2)	0.28209 (11)	0.0542 (7)	
H6	0.2121	0.2943	0.3217	0.065*	
N7	0.1565 (3)	0.2589 (2)	0.18135 (11)	0.0459 (6)	
N8	0.1743 (3)	0.2251 (2)	0.11665 (11)	0.0463 (6)	
C1	0.0902 (4)	1.0862 (3)	0.70951 (15)	0.0566 (9)	
C2	0.1044 (4)	1.1357 (3)	0.65067 (14)	0.0463 (8)	
C3	0.0605 (4)	1.2523 (3)	0.64682 (16)	0.0607 (9)	
H3A	0.0690	1.2880	0.6081	0.073*	
C4	0.0045 (5)	1.3162 (4)	0.6992 (2)	0.0762 (11)	
H4	−0.0224	1.3948	0.6960	0.091*	
C5	−0.0114 (5)	1.2632 (5)	0.7562 (2)	0.0841 (13)	
H5	−0.0512	1.3057	0.7913	0.101*	
C6	0.0306 (5)	1.1495 (4)	0.76173 (17)	0.0767 (12)	
H6A	0.0192	1.1141	0.8005	0.092*	
C7	0.1671 (4)	1.0702 (3)	0.59493 (14)	0.0480 (8)	
H7	0.1953	0.9918	0.5973	0.058*	
C8	0.2777 (4)	1.1187 (3)	0.43966 (14)	0.0434 (7)	
C9	0.4020 (4)	0.8808 (3)	0.34211 (15)	0.0499 (8)	
H9	0.4075	0.9231	0.3054	0.060*	
C10	0.4389 (4)	0.7564 (3)	0.33816 (14)	0.0455 (8)	
C11	0.4785 (4)	0.6972 (3)	0.28059 (15)	0.0515 (8)	
C12	0.5155 (5)	0.5792 (3)	0.27706 (19)	0.0674 (10)	
H12	0.5434	0.5420	0.2383	0.081*	
C13	0.5112 (5)	0.5166 (4)	0.3307 (2)	0.0785 (12)	
H13	0.5351	0.4364	0.3283	0.094*	
C14	0.4719 (5)	0.5714 (3)	0.3881 (2)	0.0758 (11)	
H14	0.4688	0.5284	0.4245	0.091*	
C15	0.4369 (4)	0.6898 (3)	0.39182 (16)	0.0596 (9)	
H15	0.4114	0.7265	0.4311	0.072*	
C16	−0.0109 (4)	0.4711 (3)	0.11266 (15)	0.0498 (8)	
C17	−0.0726 (4)	0.3890 (3)	0.15659 (14)	0.0444 (8)	
C18	−0.2529 (5)	0.3556 (3)	0.16078 (16)	0.0599 (9)	
H18	−0.2967	0.3019	0.1907	0.072*	
C19	−0.3681 (5)	0.4010 (4)	0.12104 (19)	0.0719 (11)	
H19	−0.4891	0.3765	0.1235	0.086*	
C20	−0.3039 (6)	0.4819 (4)	0.07809 (18)	0.0759 (12)	
H20	−0.3819	0.5130	0.0516	0.091*	
C21	−0.1245 (5)	0.5180 (3)	0.07351 (16)	0.0667 (10)	
H21	−0.0814	0.5735	0.0443	0.080*	
C22	0.0461 (4)	0.3399 (3)	0.20069 (14)	0.0443 (8)	
C23	0.2465 (4)	0.2311 (3)	0.23466 (14)	0.0460 (8)	
C24	0.2022 (4)	0.1161 (3)	0.10273 (14)	0.0486 (8)	
H24	0.2049	0.0624	0.1346	0.058*	
C25	0.2301 (4)	0.0749 (3)	0.03672 (14)	0.0479 (8)	
C26	0.2425 (4)	−0.0464 (3)	0.01809 (15)	0.0561 (9)	
C27	0.2720 (5)	−0.0836 (4)	−0.0444 (2)	0.0790 (12)	
H27	0.2803	−0.1657	−0.0561	0.095*	
C28	0.2890 (6)	0.0012 (5)	−0.08861 (19)	0.0903 (14)	
H28	0.3108	−0.0233	−0.1306	0.108*	
C29	0.2744 (6)	0.1223 (5)	−0.07193 (18)	0.0872 (13)	
H29	0.2839	0.1789	−0.1027	0.105*	
C30	0.2458 (5)	0.1594 (3)	−0.00994 (15)	0.0651 (10)	
H30	0.2366	0.2415	0.0013	0.078*	
C31	0.1607 (8)	0.5962 (6)	0.5595 (4)	0.192 (4)	
H31A	0.0341	0.5822	0.5574	0.288*	
H31B	0.2023	0.5618	0.5966	0.288*	
H31C	0.2014	0.5572	0.5217	0.288*	
C32	0.2229 (7)	0.7192 (6)	0.5636 (3)	0.137 (2)	
H32A	0.3501	0.7290	0.5621	0.164*	
H32B	0.1986	0.7524	0.6060	0.164*	

### Atomic displacement parameters (Å^2^)

**Table:**

	*U*^11^	*U*^22^	*U*^33^	*U*^12^	*U*^13^	*U*^23^
S1	0.0842 (7)	0.0466 (5)	0.0462 (5)	0.0223 (5)	0.0086 (4)	0.0072 (4)
S2	0.0711 (6)	0.0711 (6)	0.0526 (5)	0.0330 (5)	0.0123 (5)	0.0211 (5)
Cl1	0.1144 (9)	0.0774 (7)	0.0609 (6)	0.0123 (6)	0.0128 (6)	0.0226 (5)
Cl2	0.0862 (7)	0.0867 (7)	0.0473 (5)	0.0142 (6)	0.0118 (5)	−0.0027 (5)
Cl3	0.0576 (6)	0.0654 (6)	0.0864 (7)	0.0088 (5)	0.0044 (5)	0.0266 (5)
Cl4	0.1162 (9)	0.0531 (6)	0.0935 (8)	0.0272 (6)	0.0055 (6)	0.0064 (5)
O1	0.086 (2)	0.0733 (18)	0.099 (2)	0.0229 (17)	0.0189 (17)	0.0211 (16)
N1	0.0572 (18)	0.0483 (16)	0.0421 (15)	0.0134 (13)	0.0085 (13)	0.0011 (13)
N2	0.0617 (18)	0.0441 (15)	0.0402 (14)	0.0176 (13)	0.0103 (13)	0.0080 (12)
N3	0.0725 (19)	0.0443 (16)	0.0391 (14)	0.0180 (14)	0.0112 (13)	0.0080 (12)
N4	0.0585 (18)	0.0404 (15)	0.0442 (15)	0.0139 (13)	0.0096 (13)	0.0052 (12)
N5	0.071 (2)	0.0499 (17)	0.0428 (15)	0.0239 (15)	0.0053 (14)	0.0018 (13)
N6	0.073 (2)	0.0553 (17)	0.0381 (15)	0.0198 (15)	0.0002 (14)	0.0066 (13)
N7	0.0568 (17)	0.0475 (16)	0.0378 (14)	0.0186 (13)	0.0076 (13)	0.0071 (12)
N8	0.0552 (17)	0.0499 (17)	0.0374 (14)	0.0180 (13)	0.0080 (12)	0.0049 (12)
C1	0.049 (2)	0.070 (2)	0.0462 (19)	0.0005 (18)	0.0070 (16)	−0.0004 (18)
C2	0.0424 (19)	0.050 (2)	0.0436 (18)	0.0020 (16)	0.0050 (15)	−0.0025 (16)
C3	0.059 (2)	0.066 (2)	0.056 (2)	0.0138 (19)	0.0020 (18)	−0.0050 (19)
C4	0.069 (3)	0.074 (3)	0.084 (3)	0.021 (2)	0.003 (2)	−0.021 (2)
C5	0.072 (3)	0.108 (4)	0.068 (3)	0.016 (3)	0.019 (2)	−0.027 (3)
C6	0.077 (3)	0.101 (3)	0.046 (2)	0.002 (3)	0.016 (2)	−0.005 (2)
C7	0.052 (2)	0.0485 (19)	0.0436 (18)	0.0088 (16)	0.0053 (15)	0.0021 (16)
C8	0.0454 (19)	0.0458 (19)	0.0402 (17)	0.0115 (15)	0.0025 (14)	0.0045 (15)
C9	0.059 (2)	0.047 (2)	0.0444 (18)	0.0098 (17)	0.0050 (16)	0.0072 (16)
C10	0.047 (2)	0.0381 (18)	0.0503 (19)	0.0071 (15)	0.0035 (15)	−0.0002 (15)
C11	0.045 (2)	0.053 (2)	0.055 (2)	0.0086 (16)	−0.0013 (16)	−0.0042 (17)
C12	0.065 (3)	0.058 (2)	0.077 (3)	0.016 (2)	0.001 (2)	−0.017 (2)
C13	0.081 (3)	0.048 (2)	0.107 (3)	0.021 (2)	−0.008 (3)	−0.004 (2)
C14	0.092 (3)	0.054 (2)	0.083 (3)	0.019 (2)	−0.007 (2)	0.013 (2)
C15	0.070 (3)	0.049 (2)	0.061 (2)	0.0120 (18)	0.0052 (19)	0.0037 (18)
C16	0.050 (2)	0.050 (2)	0.0506 (19)	0.0145 (16)	−0.0002 (16)	−0.0009 (16)
C17	0.055 (2)	0.0372 (17)	0.0430 (17)	0.0168 (16)	0.0058 (15)	−0.0046 (14)
C18	0.060 (2)	0.059 (2)	0.063 (2)	0.0171 (19)	0.0163 (19)	−0.0038 (18)
C19	0.052 (2)	0.081 (3)	0.084 (3)	0.023 (2)	0.003 (2)	−0.007 (2)
C20	0.071 (3)	0.096 (3)	0.069 (3)	0.042 (3)	−0.009 (2)	0.003 (2)
C21	0.072 (3)	0.078 (3)	0.058 (2)	0.031 (2)	0.002 (2)	0.018 (2)
C22	0.052 (2)	0.0393 (18)	0.0441 (18)	0.0124 (15)	0.0102 (15)	0.0049 (15)
C23	0.057 (2)	0.0432 (18)	0.0390 (17)	0.0106 (16)	0.0048 (15)	0.0058 (15)
C24	0.060 (2)	0.047 (2)	0.0419 (18)	0.0171 (17)	0.0053 (16)	0.0078 (15)
C25	0.049 (2)	0.055 (2)	0.0431 (18)	0.0173 (16)	0.0067 (15)	0.0044 (16)
C26	0.056 (2)	0.059 (2)	0.054 (2)	0.0198 (18)	−0.0012 (17)	−0.0064 (18)
C27	0.081 (3)	0.083 (3)	0.073 (3)	0.030 (2)	0.003 (2)	−0.025 (2)
C28	0.092 (3)	0.130 (4)	0.048 (2)	0.031 (3)	0.006 (2)	−0.023 (3)
C29	0.106 (4)	0.114 (4)	0.048 (2)	0.030 (3)	0.021 (2)	0.015 (2)
C30	0.078 (3)	0.073 (3)	0.051 (2)	0.027 (2)	0.0156 (19)	0.0117 (19)
C31	0.122 (5)	0.109 (5)	0.359 (11)	0.040 (4)	−0.016 (6)	0.079 (6)
C32	0.084 (4)	0.125 (5)	0.211 (7)	0.027 (4)	−0.004 (4)	0.056 (5)

### Geometric parameters (Å, °)

**Table:**

S1—C8	1.682 (3)	C10—C11	1.394 (4)
S2—C23	1.669 (3)	C11—C12	1.373 (4)
Cl1—C1	1.735 (4)	C12—C13	1.364 (5)
Cl2—C11	1.739 (3)	C12—H12	0.93
Cl3—C16	1.730 (3)	C13—C14	1.370 (5)
Cl4—C26	1.732 (3)	C13—H13	0.93
O1—C32	1.380 (5)	C14—C15	1.371 (4)
O1—H1	0.82	C14—H14	0.93
N1—C7	1.267 (3)	C15—H15	0.93
N1—N2	1.373 (3)	C16—C21	1.371 (4)
N2—C8	1.341 (3)	C16—C17	1.383 (4)
N2—H2	0.86	C17—C18	1.383 (4)
N3—C8	1.338 (3)	C17—C22	1.474 (4)
N3—N4	1.372 (3)	C18—C19	1.378 (5)
N3—H3	0.86	C18—H18	0.93
N4—C9	1.279 (3)	C19—C20	1.365 (5)
N5—C22	1.294 (3)	C19—H19	0.93
N5—N6	1.370 (3)	C20—C21	1.381 (5)
N6—C23	1.340 (3)	C20—H20	0.93
N6—H6	0.86	C21—H21	0.93
N7—C23	1.378 (3)	C24—C25	1.457 (4)
N7—C22	1.382 (3)	C24—H24	0.93
N7—N8	1.396 (3)	C25—C26	1.380 (4)
N8—C24	1.267 (3)	C25—C30	1.398 (4)
C1—C6	1.385 (4)	C26—C27	1.379 (5)
C1—C2	1.389 (4)	C27—C28	1.363 (5)
C2—C3	1.390 (4)	C27—H27	0.93
C2—C7	1.463 (4)	C28—C29	1.374 (6)
C3—C4	1.379 (4)	C28—H28	0.93
C3—H3A	0.93	C29—C30	1.368 (5)
C4—C5	1.372 (5)	C29—H29	0.93
C4—H4	0.93	C30—H30	0.93
C5—C6	1.358 (5)	C31—C32	1.350 (7)
C5—H5	0.93	C31—H31A	0.96
C6—H6A	0.93	C31—H31B	0.96
C7—H7	0.93	C31—H31C	0.96
C9—C10	1.441 (4)	C32—H32A	0.97
C9—H9	0.93	C32—H32B	0.97
C10—C15	1.391 (4)		
			
C32—O1—H1	109.5	C14—C15—H15	119.3
C7—N1—N2	116.9 (2)	C10—C15—H15	119.3
C8—N2—N1	117.8 (2)	C21—C16—C17	121.3 (3)
C8—N2—H2	121.1	C21—C16—Cl3	118.6 (3)
N1—N2—H2	121.1	C17—C16—Cl3	120.0 (2)
C8—N3—N4	121.6 (2)	C16—C17—C18	118.5 (3)
C8—N3—H3	119.2	C16—C17—C22	122.6 (3)
N4—N3—H3	119.2	C18—C17—C22	118.9 (3)
C9—N4—N3	114.8 (2)	C19—C18—C17	120.7 (3)
C22—N5—N6	103.9 (2)	C19—C18—H18	119.7
C23—N6—N5	114.6 (2)	C17—C18—H18	119.7
C23—N6—H6	122.7	C20—C19—C18	119.7 (4)
N5—N6—H6	122.7	C20—C19—H19	120.1
C23—N7—C22	108.6 (2)	C18—C19—H19	120.1
C23—N7—N8	129.7 (2)	C19—C20—C21	120.8 (4)
C22—N7—N8	121.5 (2)	C19—C20—H20	119.6
C24—N8—N7	116.3 (2)	C21—C20—H20	119.6
C6—C1—C2	121.3 (3)	C16—C21—C20	119.1 (3)
C6—C1—Cl1	118.1 (3)	C16—C21—H21	120.5
C2—C1—Cl1	120.6 (2)	C20—C21—H21	120.5
C3—C2—C1	117.3 (3)	N5—C22—N7	110.7 (3)
C3—C2—C7	120.6 (3)	N5—C22—C17	125.3 (3)
C1—C2—C7	122.1 (3)	N7—C22—C17	123.9 (3)
C4—C3—C2	121.3 (3)	N6—C23—N7	102.1 (2)
C4—C3—H3A	119.3	N6—C23—S2	127.3 (2)
C2—C3—H3A	119.3	N7—C23—S2	130.5 (2)
C5—C4—C3	119.6 (4)	N8—C24—C25	119.9 (3)
C5—C4—H4	120.2	N8—C24—H24	120.0
C3—C4—H4	120.2	C25—C24—H24	120.0
C6—C5—C4	120.6 (4)	C26—C25—C30	118.0 (3)
C6—C5—H5	119.7	C26—C25—C24	122.0 (3)
C4—C5—H5	119.7	C30—C25—C24	120.0 (3)
C5—C6—C1	119.8 (4)	C27—C26—C25	121.4 (3)
C5—C6—H6A	120.1	C27—C26—Cl4	118.0 (3)
C1—C6—H6A	120.1	C25—C26—Cl4	120.6 (2)
N1—C7—C2	118.5 (3)	C28—C27—C26	119.3 (4)
N1—C7—H7	120.8	C28—C27—H27	120.3
C2—C7—H7	120.8	C26—C27—H27	120.3
N3—C8—N2	116.5 (3)	C27—C28—C29	120.8 (4)
N3—C8—S1	119.8 (2)	C27—C28—H28	119.6
N2—C8—S1	123.7 (2)	C29—C28—H28	119.6
N4—C9—C10	122.2 (3)	C30—C29—C28	119.9 (4)
N4—C9—H9	118.9	C30—C29—H29	120.0
C10—C9—H9	118.9	C28—C29—H29	120.0
C15—C10—C11	116.9 (3)	C29—C30—C25	120.6 (4)
C15—C10—C9	121.4 (3)	C29—C30—H30	119.7
C11—C10—C9	121.7 (3)	C25—C30—H30	119.7
C12—C11—C10	121.6 (3)	C32—C31—H31A	109.5
C12—C11—Cl2	118.3 (3)	C32—C31—H31B	109.5
C10—C11—Cl2	120.1 (2)	H31A—C31—H31B	109.5
C13—C12—C11	119.8 (3)	C32—C31—H31C	109.5
C13—C12—H12	120.1	H31A—C31—H31C	109.5
C11—C12—H12	120.1	H31B—C31—H31C	109.5
C12—C13—C14	120.3 (4)	C31—C32—O1	122.3 (6)
C12—C13—H13	119.8	C31—C32—H32A	106.8
C14—C13—H13	119.8	O1—C32—H32A	106.8
C13—C14—C15	120.0 (4)	C31—C32—H32B	106.8
C13—C14—H14	120.0	O1—C32—H32B	106.8
C15—C14—H14	120.0	H32A—C32—H32B	106.6
C14—C15—C10	121.4 (3)		
			
C7—N1—N2—C8	−173.4 (3)	C21—C16—C17—C22	−178.1 (3)
C8—N3—N4—C9	174.7 (3)	Cl3—C16—C17—C22	−1.6 (4)
C22—N5—N6—C23	−1.1 (4)	C16—C17—C18—C19	1.4 (5)
C23—N7—N8—C24	38.7 (4)	C22—C17—C18—C19	179.0 (3)
C22—N7—N8—C24	−147.1 (3)	C17—C18—C19—C20	−1.4 (5)
C6—C1—C2—C3	1.5 (5)	C18—C19—C20—C21	0.6 (6)
Cl1—C1—C2—C3	−176.9 (2)	C17—C16—C21—C20	−0.3 (5)
C6—C1—C2—C7	−180.0 (3)	Cl3—C16—C21—C20	−176.8 (3)
Cl1—C1—C2—C7	1.6 (4)	C19—C20—C21—C16	0.3 (6)
C1—C2—C3—C4	−0.2 (5)	N6—N5—C22—N7	2.1 (3)
C7—C2—C3—C4	−178.8 (3)	N6—N5—C22—C17	178.9 (3)
C2—C3—C4—C5	−1.1 (6)	C23—N7—C22—N5	−2.5 (4)
C3—C4—C5—C6	1.2 (6)	N8—N7—C22—N5	−177.7 (3)
C4—C5—C6—C1	0.1 (6)	C23—N7—C22—C17	−179.3 (3)
C2—C1—C6—C5	−1.5 (6)	N8—N7—C22—C17	5.5 (4)
Cl1—C1—C6—C5	176.9 (3)	C16—C17—C22—N5	115.1 (4)
N2—N1—C7—C2	178.9 (3)	C18—C17—C22—N5	−62.5 (4)
C3—C2—C7—N1	0.7 (5)	C16—C17—C22—N7	−68.6 (4)
C1—C2—C7—N1	−177.7 (3)	C18—C17—C22—N7	113.9 (3)
N4—N3—C8—N2	−2.2 (4)	N5—N6—C23—N7	−0.4 (3)
N4—N3—C8—S1	176.7 (2)	N5—N6—C23—S2	176.6 (2)
N1—N2—C8—N3	−178.5 (3)	C22—N7—C23—N6	1.6 (3)
N1—N2—C8—S1	2.6 (4)	N8—N7—C23—N6	176.4 (3)
N3—N4—C9—C10	178.8 (3)	C22—N7—C23—S2	−175.2 (3)
N4—C9—C10—C15	−1.7 (5)	N8—N7—C23—S2	−0.5 (5)
N4—C9—C10—C11	178.5 (3)	N7—N8—C24—C25	−177.3 (3)
C15—C10—C11—C12	−0.6 (5)	N8—C24—C25—C26	−173.1 (3)
C9—C10—C11—C12	179.3 (3)	N8—C24—C25—C30	7.1 (5)
C15—C10—C11—Cl2	−179.8 (2)	C30—C25—C26—C27	1.0 (5)
C9—C10—C11—Cl2	0.1 (4)	C24—C25—C26—C27	−178.8 (3)
C10—C11—C12—C13	1.0 (5)	C30—C25—C26—Cl4	−179.9 (3)
Cl2—C11—C12—C13	−179.8 (3)	C24—C25—C26—Cl4	0.3 (4)
C11—C12—C13—C14	−0.6 (6)	C25—C26—C27—C28	−0.2 (6)
C12—C13—C14—C15	−0.1 (6)	Cl4—C26—C27—C28	−179.3 (3)
C13—C14—C15—C10	0.6 (6)	C26—C27—C28—C29	−1.0 (7)
C11—C10—C15—C14	−0.2 (5)	C27—C28—C29—C30	1.3 (7)
C9—C10—C15—C14	179.9 (3)	C28—C29—C30—C25	−0.4 (6)
C21—C16—C17—C18	−0.5 (5)	C26—C25—C30—C29	−0.7 (5)
Cl3—C16—C17—C18	175.9 (2)	C24—C25—C30—C29	179.1 (3)

### Hydrogen-bond geometry (Å, °)

**Table:**

*D*—H···*A*	*D*—H	H···*A*	*D*···*A*	*D*—H···*A*
N2—H2···O1	0.86	2.20	2.978 (4)	150
N6—H6···S1^i^	0.86	2.41	3.261 (3)	170
N3—H3···S2^ii^	0.86	2.56	3.410 (2)	169
O1—H1···S1^iii^	0.82	2.65	3.413 (3)	156

Symmetry codes: (i) *x*, *y*−1, *z*; (ii) *x*, *y*+1, *z*; (iii) −*x*, −*y*+2, −*z*+1.

## References

[bb1] Bacci, A., Carcelli, M., Pelagatti, P., Pelizzi, G., Rodriguez-Arguelles, M. C., Rogolino, D., Solinas, C. & Zani, F. (2005). *J. Inorg. Biochem.* **99**, 397–408. 10.1016/j.jinorgbio.2004.10.008 15621271

[bb2] Bruker (2005). *SMART* and *SAINT* Bruker AXS Inc., Madison, Wisconsin, USA.

[bb3] Liang, F.-Z. (2003). *J. Shandong Normal Univ. (Nat. Sci.)*, **18**, 50–51.

[bb4] Ren, Y. P., Dai, R. B., Wang, L. F. & Wu, J. G. (1999). *Synth. Commun.* **29**, 613–617.

[bb5] Sen, A. K., Singh, R. N., Handa, R. N., Dubey, S. N. & Squattrito, P. J. (1998). *J. Mol. Struct.* **470**, 61–69.

[bb6] Sheldrick, G. M. (1996). *SADABS* University of Göttingen, Germany

[bb7] Sheldrick, G. M. (2008). *Acta Cryst.* A**64**, 112–122. 10.1107/S0108767307043930 18156677

[bb8] Xia, H.-T., Liu, Y.-F., Yang, S.-P. & Wang, D.-Q. (2007). *Acta Cryst.* E**63**, o40–o41.

[bb9] Yang, J. G. & Pan, F. Y. (2005). *Chin. J. Struct. Chem.* **24**, 1403–1407.

